# Construction Notes

**Published:** 1893-12-02

**Authors:** 


### CONSTRUCTION NOTES.

### Sunderland Eye Infirmary.

This building has been erected from the plans of Mr. Donald, of South Shields, the contract work having been carried out by Messrs. D. and J. Ranken, of Sunderland, at a cost bordering on £7,000, all of which money, with the exception of about £800, has now been raised. The interior arrangements show 14 rooms on the basement, the most commodious being the board-room, and the general waiting-room adjoining it. The culinary section of the building has been fitted up with the latest modern appliances. Leading up from the entrance hall is a staircase of solid polished oak, while the corridors on each storey are paved with red tiles. The wards, which are four in number, afford accommodation for sixteen patients. The whole of the attics have been built with a view of conversion into bed-rooms should this become necessary.

### New Hospital for Women at Nottingham.

This hospital, opened on October 12th, provides accommodation for twenty-five or thirty patients; the premises, which have undergone a complete alteration, were formerly used as Inland Revenue offices, but three years ago were purchased by the Women's Hospital Committee for the above purpose. The alteration on the offices was in the first case effected by piercing the partition walls which separated the two houses. There is a spacious basement, which includes kitchens, larders, laundry, and drying-stove, and on the ground floor there is a board-room, matron's room, an out-patient's department, with an entrance entirely separate from the other part of the hospital. On the first floor there are wards, an operating-room, a bathroom, a ward kitchen, water-closet, and a hospital sink, whilst the second floor affords the same accommodation. A hoist has been erected in the centre of the building, running from the basement to the second floor, which is for the conveyance of food, coal, &c. The third floor is devoted to the convenience of servants and nurses; for the latter a large sitting-room and bath-room are included. The building has been fitted throughout with the latest sanitary improvements. The work was carried out by Mr. Mersom, builder, and Messrs. Humphrey and Co., of Nottingham, from the architectural plans of Messrs. Evans and Jolly, also of Nottingham.

### Permanent Hospital, Johannesburg, South Africa.

Mr. A. H. Reed, F.R.I.B.A., is the architect of this permanent hospital which is fitted with every modern appliance for the convenience and comfort of the patients and nursing staff. The total bed accommodation is for about 120 whites and 90 natives. the latter being kept in a separate building. For the ventilation of the separate wards a system of natural ventilation has been adopted throughout the building by regulating the inlet and outlet vents. The wards are fitted with Sheringham's ventilators. The sanitation is necessarily of a primitive nature, but is nearly perfect in its way. The whole of the building is lighted with oil. New native wards are in course of construction, and the contagious wards, separated from the main buildings, are completed. The total expenditure up to date is £53,000 on the buildings and furniture.

